# Systemic Revealing Pharmacological Signalling Pathway Networks in the Hippocampus of Ischaemia-Reperfusion Mice Treated with Baicalin

**DOI:** 10.1155/2013/630723

**Published:** 2013-11-17

**Authors:** Haixia Li, Yingying Zhang, Yanan Yu, Bing Li, YinYing Chen, Hongli Wu, Jingtao Wang, Jun Li, Xingjiang Xiong, Qiongyong He, Jinzhou Tian, Zhong Wang, Jie Wang

**Affiliations:** ^1^Department of Cardiology, Guang'anmen Hospital, China Academy of Chinese Medical Sciences, Beixiange 5, Xicheng District, Beijing 100053, China; ^2^Institute of Basic Research in Clinical Medicine, China Academy of Chinese Medical Sciences, Dongzhimen, Beijing 100700, China; ^3^Geriatric Department, Dongzhimen Hospital Affiliated to Beijing University of Chinese Medicine, Dongzhimen, Beijing 100007, China

## Abstract

*Background*. Baicalin (BA) exhibits ill understood neuroprotective, anti-inflammatory, and antioxidative effects in brain injury. *Objective*. To identify the differential network pathways associated with BA-related biological effects. *Methods*. MCAO-induced mice received BA 5 mg/Kg (BA group). Controls received vehicle only. Following ischaemia-reperfusion, ArrayTrack analysed the whole genome microarray of hippocampal genes, and MetaCore analysed differentially expressed genes. *Results*. Four reversing pathways were common to BA and controls, but only 6 were in the top 10 for BA. Three of the top 5 signalling pathways in controls were not observed in BA. BA treatment made absent 3 pathways of the top 5 signalling pathways from the top 5 in controls. There were 2 reversing pathways between controls and BA that showed altered gene expression. Controls had 6 networks associated with cerebral ischaemia. After BA treatment, 9 networks were associated with cerebral ischaemia. Enrichment analysis identified 10 significant biological processes in BA and controls. Of the 10 most significant molecular functions, 7 were common to BA and controls, and only 3 occurred in BA. BA and controls had 7 significant cellular components. *Conclusions.* This study showed that the clinical effectiveness of BA was based on the complementary effects of multiple pathways and networks.

## 1. Introduction

The biochemical and molecular mechanisms underlying cerebral ischaemia, such as reperfusion injuries (common in stroke patients), alterations in multiple genes, proteins, and mechanistic pathways, cumulatively lead to progressive neurological damage and cell death [1]. Cerebral ischaemia mechanisms involve mitogen-activated protein kinases (MAPKs), phosphoinositide 3-kinase/protein kinase B, nuclear factor kappa-light-chain-enhancer of activated B cells, cAMP response element-binding protein, Janus kinase-signal transducer and activator of transcription (STAT) pathway, WNT/beta-catenin pathway, and excessive inflammatory response [2–7]. Elucidating details of these complex mechanisms are difficult despite recent improvements in research technologies, such as reverse pharmacology. A better understanding is however critical to facilitate development of novel therapeutic agents.

Baicalin (5,6-dihydroxy-7-O-glucuronide flavone) (BA) is a flavonoid compound, extracted from the dry dicotyledonous skullcap root commonly used in traditional Chinese and clinical medicine to treat stroke [1]. BA exerts neuroprotective, anti-inflammatory, and antioxidative effects in a variety of animal models of brain injury [8–14], but its mechanism of action is not well understood. Initial studies of BA's mechanism of action focused on differential gene expression. By identifying many potentially up- or downregulated candidate genes, these studies produced complex and disparate mechanistic data. Genes studied included those involved in TLR2/4 signalling [15, 16], apoptosis [1, 12, 17], reactive oxygen species scavenging [12], GABAergic signalling [11], calcium signalling, and tight junction proteins in the blood-brain barrier [17, 18].

Several contemporary analytical tools have been developed to determine trends and to assimilate and visualize molecular interaction data, such as Ingenuity Pathway Analysis (IPA) [19] and the Kyoto Encyclopaedia of Genes and Genomes (KEGG) [20] databases. These tools, coupled with computerized databases, allow a better understanding of the gene interactions that cumulatively affect important biological pathways and have analysed the influence of BA on gene molecular functions and network path functions. In a study of a cDNA microarray of 374 genes, ArrayTrack and IPA pathway analysis showed that the genes influenced by BA participated in calcium regulation, cell signal transduction, cell proliferation, and antiapoptotic mechanisms [21]. In a study of a microarray of 16464 genes, ArrayTrack and KEGG pathway analysis showed that BA affected expression of 361 distinct genes was associated with activation of 76 pathways, including those regulating extracellular matrix receptors and ATP-binding cassette transporters [22]. Despite increases in available raw data, limited computing power and small databases have reduced the ability of research efforts to establish associations between numerous and complex cellular mechanisms following ischemic events involving many cell types and pathways. An analysis of the full array of mechanisms that occur following ischaemia requires more advanced software solutions, such as larger datasets and more advanced data retrieval methods. GeneGo MetaCore software [23] uses a unique, proprietary, high-quality, manually curated database of human protein-protein, protein-DNA, and protein-RNA interactions. Unlike previous software systems, MetaCore integrates information on signalling and metabolic pathways and the effects of bioactive molecules [24]. Another benefit is that data are presented within an intuitive graphical model, which allows transcriptomics data to be visualized [25]. MetaCore software was used to identify the differential pathway networks of BA in a rodent model of cerebral ischaemia-reperfusion injury. A microarray of 16,463 genes, analyzed with combined ArrayTrack and MetaCore systems, identified differential pathway networks from gene expression profiles in the hippocampus of mice with cerebral ischaemia following BA administration.

## 2. Methods

### 2.1. Animal Subjects

There were 144 healthy, specific pathogen-free, adult male Kunming mice aged 12 weeks of age and weighing 38 to 48 g. They were housed at 25°C with a 12-hour light/dark cycle and randomly divided into 3 groups of 48 mice (BA group, vehicle group, and sham group). Animal use protocols were reviewed and approved by the Ethics Review Committee for Animal Experimentation of the China Academy of Chinese Medical Sciences, and all animal experiments were conducted in accordance with the Prevention of Cruelty to Animals Act of 1986 and National Institute of Health guidelines for care and use of experimental laboratory animals.

### 2.2. Middle Cerebral Artery Occlusion

For the BA and vehicle groups, mice were anaesthetized with 2% napental (4 mg/Kg, intraperitoneal) then underwent surgery to induce middle cerebral artery occlusion. The middle cerebral artery was ligated with an intraluminal filament for 1.5 hours and then reperfused for 24 hours. For the sham group, the external carotid artery was surgically prepared for insertion of the filament, but no filament was inserted. During the experimental procedures, blood pressure, blood gas level, and glucose levels were monitored. Rectal temperature was maintained at 37.0 to 37.5°C with a heating pad, and body temperature was maintained at 37°C with a thermostatically controlled infrared lamp. Brain temperature was maintained at 36 to 37°C and monitored with a 29-gauge thermocouple in the right corpus striatum and a temperature-regulating lamp. An electroencephalogram was taken to ensure isoelectricity during the ischemic period. Operational success was determined based on infarct volume and subsequent mouse behaviour.

### 2.3. Drug Administration

BA group mice were administered 5 mg/Kg BA, dissolved in 0.9% normal saline immediately prior to use, by injection into the tail vein 1.5 hours after focal cerebral ischaemia induction. Vehicle and sham group mice were administered only 0.9% normal saline (2 mL/Kg). All BA preparations were standard, validated using fingerprint chromatography, and obtained from the China Natural Institute for the Control of Pharmaceutical and Biological Products or the Beijing University of Traditional Chinese Medicine.

### 2.4. RNA Isolation

The left hippocampus of 9 mice in each group was homogenized in TRIzol Reagent (Invitrogen, USA), and total RNA was isolated according to the manufacturer's instructions. RNA was further purified to remove genomic DNA contamination and concentrated using an RNeasy micro kit (Qiagen, Valencia, CA, USA). The RNA quality was determined from the 26S/18S ratio, using a Bioanalyzer microchip device (Agilent, Palo Alto, CA, USA).

### 2.5. Microarrays

A mouse brain array (Boao Capital, Beijing, China) and a microarray chip for the whole genome array for mice containing 16,463 oligoclones (Incyte Genomics, Santa Clara, CA, USA) were used for gene expression profiling. On each chip, duplicate clones were printed, generating 4 technical replicates per clone. A single intensity value for each clone was generated by averaging quadruplet measurements after smoothing spline normalization. All clones were verified by DNA sequencing. RNA from the vehicle group was pooled and labelled with Cy3, and RNA from other groups was labelled with Cy5. Microarrays were hybridized, washed, and scanned according to standard protocols. These procedures were repeated for each group, at least as biological triplicates and technical quadruplets.

### 2.6. Microarray Data Analysis

All experimental data were uploaded to the ArrayTrack system. Experimental analysis was based on the Minimum Information about Microarray Experiment Guidelines and the Microarray Quality Control Project, and the results were submitted to the Array Express database. All microarray data were normalized by locally weighted linear regression to reduce experimental variability (smoothing factor, 0.2; robustness iterations, 3). One-way analysis of variance and significance analysis of microarrays were used to compare means of the altered genes between the sham and vehicle groups and between BA and vehicle groups. Genes with a *P* < 0.05 and a >1.5-fold change were further analysed. An increase in expression level >1.5-fold or a decrease <0.5-fold was considered to indicate upregulation or downregulation, respectively. All statistically significant differentially expressed genes were uploaded into the GeneGo system [26] and gene identity numbers were uploaded to MetaCore to determine their associated networks. A cut-off value was set to identify molecules with significantly differentially regulated expressions, and these were labelled as Network Eligible molecules.

Networks of Network Eligible molecules were algorithmically generated based on connectivity. Right-tailed Fisher's exact test was used to calculate *P* values for the probability that each biological function assigned to a network was due to chance alone. The significance of association between these genes and the canonical pathway was measured in the following 2 ways:

1. a ratio calculated using the number of genes from the dataset that maps the pathway divided by the total number of genes that map to the canonical pathway;
2. a *P* value, calculated by Fischer's exact test, determining the probability that the association between the genes and canonical pathway was explained by chance alone. The level of statistical significance was set at *P* < 0.05. Finally, canonical pathways with *P* < 0.05 and a fold change >1.5 were screened and analyzed.

### 2.7. Network Calculation of Enrichment

Enrichment analysis is a computational method for identifying the functional distribution of genomic/proteomic expression profiles and significantly enriched functional categories [26]. This method is helpful to understand the overall functions of differentially expressed genes and so supply fundamental bioinformatics information. Biological processes, subcellular locations, and molecular function distributions of differentially expressed genes were computed using MetaCore based on Gene Ontology annotations [27], and the network distribution of selected genes was computed using MetaCore based on GeneGo network ontologies.

Auto Expand adds objects until a dense network is created, allowing neighbouring interactions and objects surrounding selected nodes to be visualized. Using the original input list, Analyze Network analyzes the network modularity for selected genes, thus creating a large network which can be subdivided it into smaller subnetworks sets. The resulting networks are evaluated and ranked according to their statistical significance (*P* values). This high trust *P* value calculation is also used to evaluate the network's relevance to Gene Ontology Biological Processes classification. The advantage of using this network algorithm is that it may find a well-connected cluster of root nodes without any predefined restrictions and thereby offer more flexibility in identifying possible connections. These interactions are then assigned to specific biological processes, cellular components, and/or molecular functions to further characterize the underlying condition and provide insight into the underlying mechanisms.

### 2.8. Statistical Analysis

Significance of enrichment and pathways was calculated using scores produced using the Expression Analysis Systematic Explorer software (National Institute of Health, USA) [28], which employs modified Fisher's exact test [29]. To calculate pathways, the statistical significance of ontological matches was calculated for the probability of a match occurring by chance, taking database size into account. Lower significance, which denoted higher ratings for matched terms, was expected as the number of genes/proteins belonging to a single process/pathway increased, with *P* < 0.1 considered statistically significant.

## 3. Results

### 3.1. Pharmacodynamic Results

BA was effective in reducing ischemic infarction volume compared with the vehicle group in our previous study (*P* < 0.05) [25].

### 3.2. Pathway Map Analysis of Altered Genes

**Figure 1:**
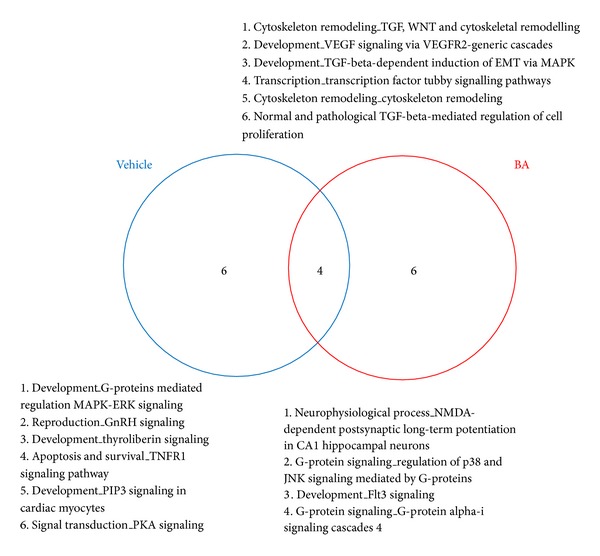
Pathway maps associated with regulated genes in both BA and vehicle groups.

**Figure 2:**
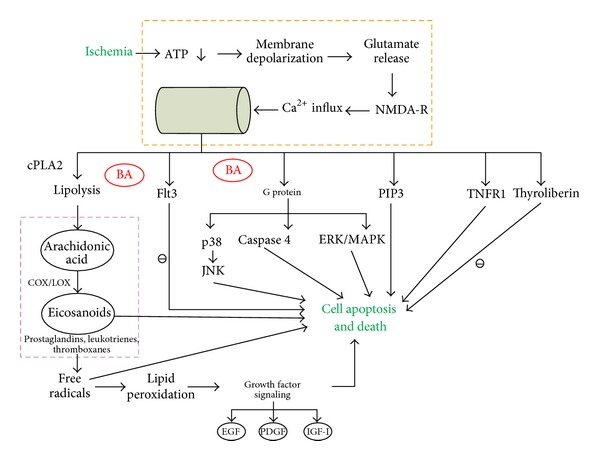
Diagram of BA action in targeting pathways involved in cell apoptosis and death.

The MetaCore pathway map analysis of the selected genes was used to identify the 10 most statistically significant pathways, based on calculated *P* values (see Supplementary Table 5A available online at http://dx.doi.org/10.1155/2013/630723). Four reversing pathways were common to both BA and vehicle groups, but only 6 pathways were among the top 10 for only the BA group: “cytoskeleton remodeling: TGF, WNT, and cytoskeletal remodeling”; “development: VEGF signalling via VEGFR2-generic cascades”; “development: TGF-beta-dependent induction of EMT via MAPK”; “transcription: transcription factor Tubby signalling pathways”; “cytoskeleton remodelling: cytoskeleton remodeling”; and “normal and pathological TGF beta-mediated regulation of cell proliferation” (Figure 1). A diagram of BA action in targeting pathways involved in cell apoptosis, and death is shown in Figure 2.

Three of the top 5 most statistically significant signalling pathways in the vehicle group were not observed in the BA group (Figure 1, Supplementary Tables 1A, 1B). Notably, in the “development: G-protein mediated regulation of MAPK-ERK signalling” pathway, 8 genes were upregulated. In the “reproduction: GnRH signalling” pathway, 9 genes were upregulated, while ATF3 was downregulated. In the “development: thyroliberin signalling” pathway, 6 genes were upregulated.

BA treatment also produced 3 of the top 5 most statistically significant signalling pathways, and these were absent from the top 5 in the vehicle group. In the “cytoskeleton remodeling: TGF, WNT, and cytoskeletal remodeling” pathway, 11 genes were upregulated. In the “development: Flt3 signalling” pathway, 8 genes were upregulated. In the “development: VEGF signalling via VEGFR2-generic cascades” pathway, 8 genes were upregulated.

**Figure 3:**
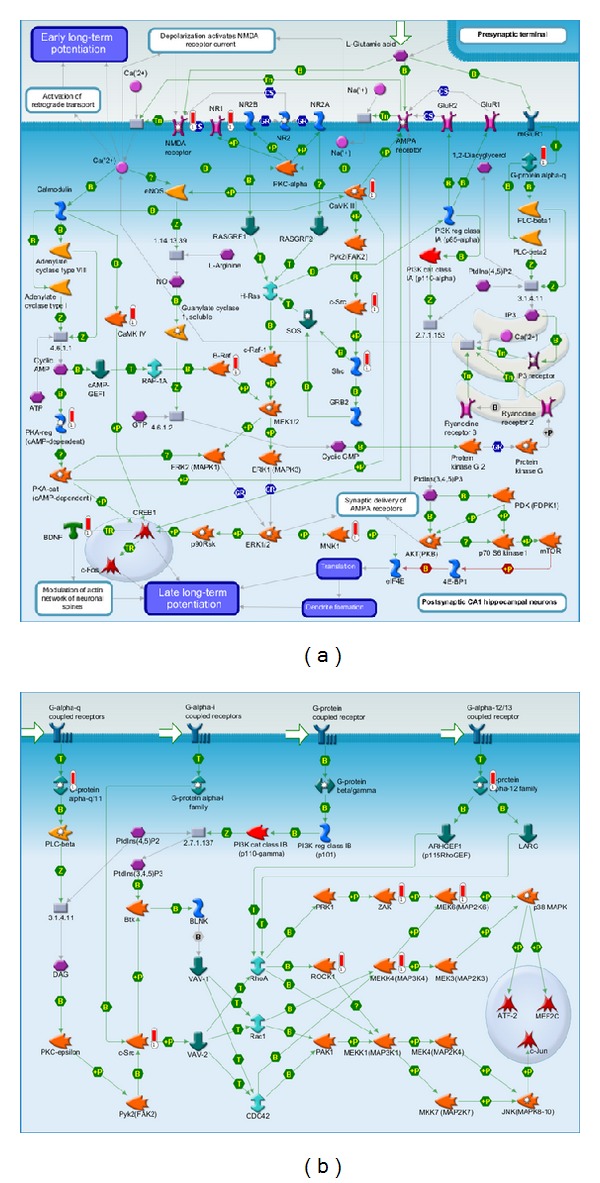
Two reversing pathways between the vehicle and baicalin groups. (a) Neurophysiological process: NMDA-dependent postsynaptic long-term potentiation in CA1 hippocampal neurons' pathway. (b) G-protein signalling: regulation of p38 and JNK signalling pathway.

There were 2 reversing pathways between the vehicle and BA groups that nonetheless showed altered gene expression. In the “neurophysiological process: NMDA-dependent postsynaptic long-term potentiation in CA1 hippocampal neurons” pathway (Figure 3(a)), the following genes were upregulated in the vehicle group: NMDA receptor, NR1, G-protein alpha-q, CaMKII, Pyk2, c-src, CaMKIV, adenylate cyclase type I, Shc, and PKA-cat. After treatment with BA, NMDA receptor, NR1, G-protein alpha-q, CaMKII, and Shc were still dominantly expressed, whereas adenylate cyclase type I and PKA-cat were not, and B-Raf, PKA-reg, BDNF, and MNK1 were upregulated. In the “G-protein signalling: regulation of p38 and JNK signalling” pathway (Figure 3(b)), the following genes were upregulated in the vehicle group: G-protein alpha-12 family, G-protein alpha-q, ARHGEF1, ZAK, c-src, and Pyk2. In the BA group, G-protein alpha-q, G-protein alpha-12 family, ZAK, and c-src were still dominantly expressed, while ARHGEF1 and Pyk2 were no longer dominantly expressed, and MEK6, ROCK1, and MEKK4 were upregulated.

### 3.3. Process Network Distribution

**Figure 4:**
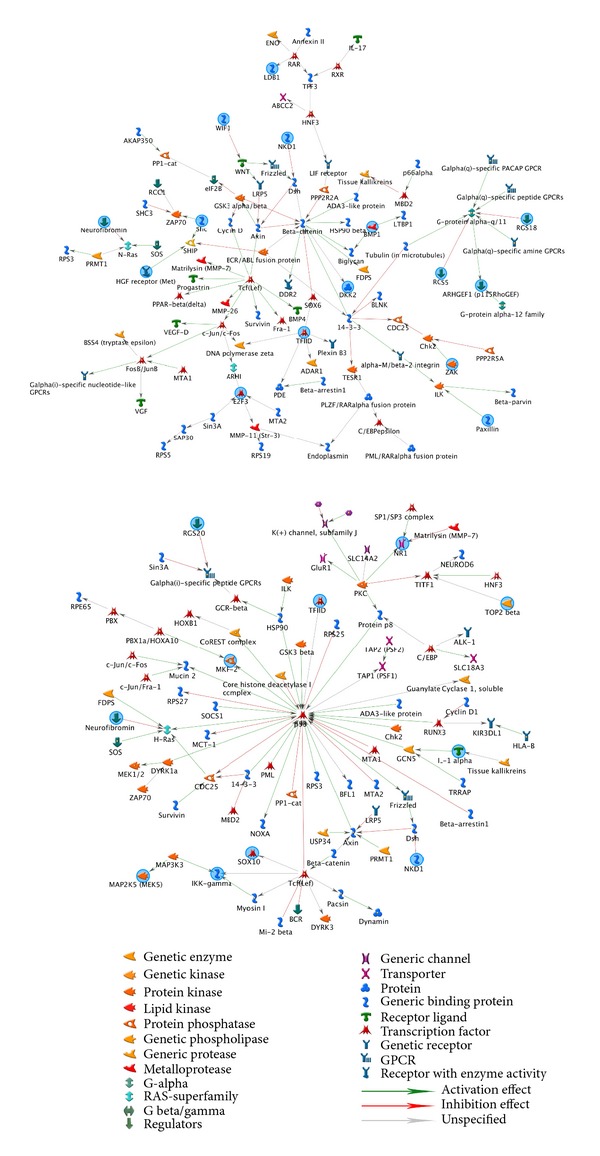
Subnetwork 1 and 5 of 6 networks in vehicle group. The subnetwork 1 primarily consisted of G-protein alpha-q, TFIID, ARHGEF1 (p115RhoGEF), Shc, and E2F3, and its major function was positive regulation of cellular process. The centre of subnetwork 5 is p53 whose major function was the regulation of cellular metabolic process.

In the vehicle group, 6 networks were associated with cerebral ischaemia, each with 150 nodes (Supplementary Table 7). The major function of subnetwork 1 was positive regulation of cellular process (Figure 4). The major function of subnetwork 2 was positive regulation of RNA metabolic process. The major function of subnetwork 3 was positive regulation of DNA-dependent transcription. The major function of subnetwork 4 was nucleotide metabolic process. The major function of subnetwork 5 was regulation of cellular metabolic process. The major function of subnetwork 6 was viral genome expression (Supplementary Figure 1, Supplementary Table 7). Subnetwork 1 and subnetwork 5 of vehicle have similar function of cellular process.

**Figure 5:**
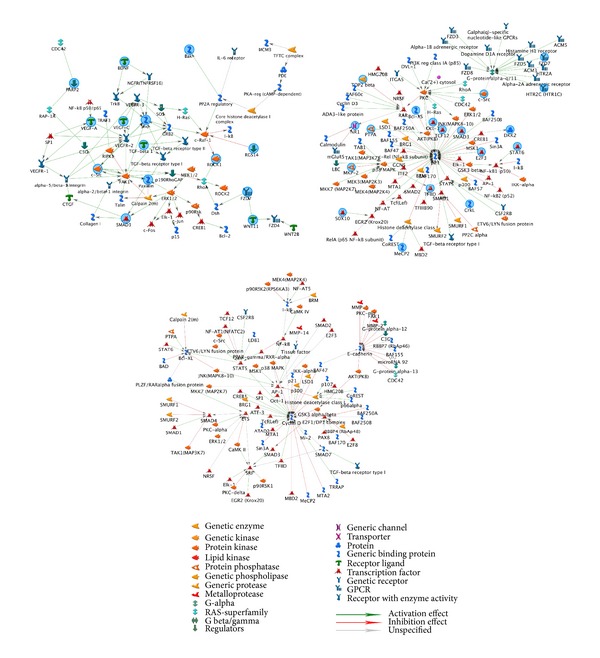
Subnetworks 1, 2, and 4 of 9 networks in baicalin group. The subnetwork 1 primarily consisted of Shc, c-src, VEGF-A, paxillin, and SMAD3, and its major function was regulation of cellular metabolic process. The centre of subnetwork 2 is p21 whose major function was positive regulation of nitrogen compound metabolic process and positive regulation of cellular biosynthetic process. The centre of subnetwork 4 is cyclin D whose major function was positive regulation of macromolecule metabolic process.

After BA treatment, there were 9 networks associated with cerebral ischaemia, with 150 nodes (Supplementary Table 6). The major function of subnetwork 1 was regulation of cellular metabolic process. The major functions of subnetwork 2 were positive regulation of the nitrogen compound metabolic process and positive regulation of the cellular biosynthetic process (Figure 5). The major functions of subnetwork 3 were intracellular signal transduction and cell surface receptor linked signalling pathway (Supplementary Figure 2). The major function of subnetwork 4 was positive regulation of macromolecule metabolic process (Figure 5). The major function of subnetwork 5 was positive regulation of macromolecule metabolic process. The major function of subnetwork 6 was positive regulation of cellular metabolic process. The major function of subnetwork 7 was regulation of molecular function. The major function of subnetwork 8 was regulation of calcium ion transport via voltage-gated calcium channel activity. The major function of subnetwork 9 was positive regulation of RNA metabolic process (Supplementary Figure 2 and Supplementary Table 6).

Sub-network 1 and sub-network 7 of BA had similar function of cellular metabolic process. Subnetwork 4 and subnetwork 5 of BA had similar function of macromolecule metabolic process.

In the BA group novel subnetwork function includes “positive regulation of the nitrogen compound metabolic process and positive regulation of the cellular biosynthetic process,” “intracellular signal transduction and cell surface receptor linked signalling pathway,” “macromolecule metabolic process,” and “regulation of calcium ion transport via voltage-gated calcium channel activity” (Supplementary Figure 2 and Supplementary Table 6).

### 3.4. Enrichment Analysis of Biological Processes in the BA and Vehicle Groups

**Figure 6:**
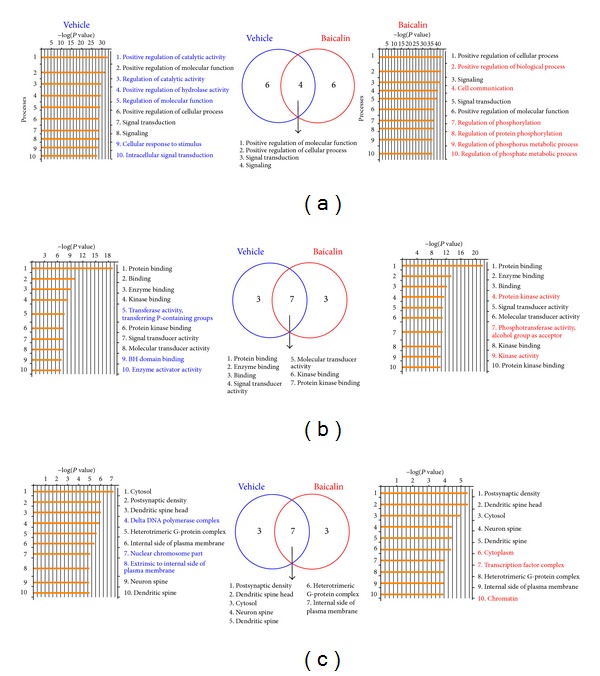
Biological process (a), molecular functions (b), and cellular components (c) associated with upregulated genes in both BA and vehicle groups.

There were 10 significant biological processes identified by enrichment analysis in the BA and vehicle groups (*P* < 0.05) (Supplementary Table 3). The biological processes “positive regulation of cellular process,” “signaling,” and “signal transduction” were among the top 10 biological processes in all groups, but “positive regulation of molecular function,” “cellular response to stimulus,” and “intracellular signal transduction” were absent from the BA group. In the BA group, novel biological processes included “positive regulation of biological process,” “cell communication,” and processes associated with phosphorylation and phosphorus/phosphate metabolism (Figure 6).

### 3.5. Distribution of Molecular Functions in the BA and Vehicle Groups

Of the 10 most significant molecular functions presented in Supplementary Table 4  (*P* < 0.05), seven molecular function categories were common to the BA and vehicle groups, and 3 categories (“phosphotransferase activity with alcohol group as acceptor,” “protein kinase binding,” and “kinase activity”) only occurred in the BA group (Figure 6).

### 3.6. Distribution of Cellular Components in the BA and Vehicle Groups

The 10 most significant cellular components (*P* < 0.05) associated with the vehicle and BA groups are shown in Supplementary Table 5. Seven cellular components were identified for both groups, and 3 (“cytoplasm,” “transcription factor complex,” and “chromatin”) appeared only in the top 10 of the BA group (Figure 6).

## 4. Discussion

Although several previous studies have investigated the effects of BA on individual gene expression, none has integrated these various findings into a coherent series of interacting functional pathways and networks [1, 9, 12, 17, 18, 18–20]. The current study is a novel combination of microarray techniques with 16463 clones analyzed with ArrayTrack and MetaCore, providing an in-depth analysis that extends the breadth of knowledge of changes in gene expression associated with cerebral ischaemia-reperfusion injury. BA pathways most apparently influenced by gene expression were associated with cytoskeleton remodelling, cellular development, VEGF signalling via VEGFR2, and TGF-beta-dependent EMT induction via MAPK. In the vehicle group by contrast, gene expression was primarily associated with G-protein mediated regulation of MAPK-ERK signalling during important developmental processes, reproductive GnRH signalling, developmental thyroliberin signalling, and TNFR1 signalling pathways associated with apoptosis and survival. Notably, gene expressions in both the BA and the vehicle groups were associated with pathways involved in NMDA-dependent postsynaptic long-term neuron-potentiation in CA1 hippocampal neurons, G-protein signalling through p38- and JNK-mediated pathways, developmental Flt3 signalling, and G-protein alpha-i signalling cascades. Furthermore, gene expression and likely mechanistic pathways influenced by BA administration were determined with great specificity. The neuroprotective effects of BA treatment relate to selective regulation of pathways, particularly p38 and JNK signalling, NMDA-dependent postsynaptic long-term potentiation, and differentially expressed gene networks, and thereby reduce apoptosis rates and downstream damage due to radical accumulation. Thus, this modern computer-based analysis allows for a more comprehensive understanding of mechanistic pathways in ischaemia-reperfusion injury and subsequent BA treatment than what was previously possible, suggesting that BA treatment induces gene expression that not only prevents apoptosis but also promotes oligodendrocyte survival and myelination signalling.

Two recent studies [24, 25] which used ArrayTrack, IPA, and KEGG-based analysis to better understand the underlying BA mechanisms of action were of limited value because they had relatively small databases. Although numerous databases are currently available for analysis of differential pathway networks, their completeness and accuracy have enormous consequences on data output quality and reliability. Thus, the current study adopted MetaCore due to its novel use of an extensive and manually curated database of human protein-protein, protein-DNA, and protein-RNA interactions, allowing a much more complete integration of signalling and metabolic pathways than previously applied sources.

There were 2 unaltered pathways between the vehicle and BA groups that showed altered gene expression: the “neurophysiological process: NMDA-dependent postsynaptic long-term potentiation in CA1 hippocampal neurons” pathway (Figure 3) and “G-protein signalling: regulation of p38 and JNK signalling” pathway (Figure 3).

Though biochemical evidence strongly indicates apoptotic processes in the postischemic period, morphological evidence interestingly does not widely support standard apoptotic process occurrence, particularly in CA1 pyramidal cells of the hippocampus [30]. BA treatment following stroke has also been demonstrateed to have antioxidative and antiapoptotic properties that may act by increasing superoxide dismutase, glutathione peroxidase and glutathione, and BDNF expression while reducing caspase-3 activity [1, 12]. Consistent with previous reports [12], BA gene expression did limit the action of apoptotic factors, such as capsase-3, but application of BA may have more profound and interesting effects on NMDA-dependent postsynaptic potentiation, associated with delayed neuronal death due to neurotoxicity [31]. Previous studies have indicated that NMDA neuroreceptor NR2A/2B aAbs are independent and sensitive serologic markers for transient ischaemia, indicative of potential elevation of damaging thrombotic (homocysteine, antiphospholipid antibodies, anticardiolipin antibodies), neurotoxic (glutamate), neurochemical markers (neuro-specific enolase, protein S100, and myelin), and peptide fragments [32]. Thus, these findings indicate that BA-induced genetic expression may potentially act to mediate ischemic damage after an initial insult by mediating cellular metabolism associated with rising neurotoxicity.

The most widely accepted theory of ischemic brain injury suggests that ischaemia causes delayed neuronal death, primarily affecting the vulnerable CA1 region of the hippocampus [33]. In fact, death of CA1 pyramidal cells has been demonstrated to be an immediate result of ischaemia [34], though it has been reported that the majority of cells at the borders of the infarct were not yet dead after 48 hours [32]. In the current study, it was found that BA acted on calcium ion (Ca^2+^)-dependent signalling cascades, shown to play a neuroprotective role in cerebral ischaemia by signalling Ca^2+^/CaM-dependent ERK activation, and this is consistent with previous findings [35]. In 1999, Lipton noticed that extracellular Ca^2+^ concentration sharply declines in the ischemic core immediately after ischaemia with a concomitant rise in extracellular potassium ions (K^+^) (*∼*70 mM) over 2 hours and returns to near normal concentration over 6 to 24 hours [31]. Thus, BA may aid mediation of harmful flux and subsequent cellular response of extracellular Ca^2+^ and K^+^ concentration. Furthermore, this hypothesis is supported by the observed impact of BA treatment on regulators of G-protein signalling 5, 6, 14, and 30 observed in the current study, which, with GPCR-kinase 2 (GRK2), may play a role in protecting receptors from overstimulation by induced desensitization [36]. Effects on GABAergic signalling associated with enhanced expression of HSP70 and phosphorylated ERK coupled with decreased levels of phosphorylated JNK and p38 have also been reported [11], consistent with the observations of the current study. Furthermore, BA may act on calcium signalling pathways, tight junction proteins of the blood-brain barrier [14, 19], and neural stem/progenitor cells [9]. Though further study is required, the potential neuroprotective role of BA via G-protein desensitization merits further study.

Consistent with previous studies, [37] the current study indicated that the G-protein network may be associated with apoptosis, as it was readily apparent without BA treatment. The current study similarly showed increasing expression of p53 network and proapoptotic Src homology 2 domain containing transforming protein (Shc) in vehicle group. Isoform p66Shc acts as a downstream target of the tumour suppressor p53 and is indispensable for stress-activated p53 to induce an increase in intracellular oxidant concentration, cytochrome c release, and apoptosis [38]. It could activate and induce apoptosis in a p53-independent manner as well [39] (Figure 5). Network including these networks and genes in vehicle group may play roles in increasing apoptosis and cell death following ischaemia-reperfusion. Conversely, treatment with BA was shown to increase the network processes for the antiapoptotic p21 and SMAD3 (Figure 6), which has been demonstrated to quantitatively affect apoptosis rates [40]. Thus, BA may act through these and other network pathways to limit apoptosis. Furthermore, cyclin D and TGF beta networks were also increased in BA treated group (Figure 6). As a substrate for SMAD3, cyclin D phosphorylated SMAD3 in a cell-cycle-dependent manner and repressed its transcriptional activity. SMAD3 has an inhibitory effect on wound healing, probably by altering the TGF-mediated chemotaxis of monocytes [40]. BA activation of the cyclinD-SMAD3-TGF beta network pathway may improve wound healing after cerebral ischaemia.

As in the use of any proprietary database, although we used Western blotting confirmed results of key proteins in the WNT3 and NF-KB pathways in different groups in our previous study [2], the study results may contain some bias based on the database used, which may not fully represent the actual biological conditions in their entirety. Furthermore, the mouse model may not completely represent the human response to ischaemia-reperfusion and BA treatment. Thus, further clinical assessments will be required to verify these results in humans [41].

## 5. Conclusion

This study used modern computer databases with extensive manually curated data sets to provide a comprehensive analysis of the pathways and networks involved in the neuroprotective effects of BA treatment for stroke. The study suggests that cerebral ischaemia may induce 10 pathways and alter 6 networks and that BA may recover ischaemia damage cells via 9 networks and 10 pathways, of which 4 are reversing. This study showed that the clinical effectiveness of BA was based on the complementary effects of multiple pathways and networks.

## Supplementary Material

Supplementary Table 3: Presents the ten most significant biological processes identified by enrichment analysis in the BA and vehicle groups (all P<0.05).
Supplementary Table 4: Shows the ten most significant molecular functions in the BA and vehicle groups (all P<0.05).
Supplementary Table 5: Presents the ten most significant cellular components associated with the BA and vehicle groups (all P<0.05).
Supplementary Table 5A: Presents the ten most statistically significant pathways, based on the MetaCore^TM^ pathway map analysis (all P<0.05).
Supplementary Table 6: Presents the nine networks associated with cerebral ischemia after BA treatment, each with 150 nodes.
Supplementary Table 7 and Supplementary Figure 1: Presents the six networks associated with cerebral ischemia in the vehicle group, each with 150 nodes.
Supplementary Figure 2: shows that sub network 3 primarily consisted of ZAK, LDB1, TCF12, WIF1, and Kallikrein 1, and that its major functions were intracellular signal transduction and cell surface receptor linked signalling pathway.
