# Supplementary material for: Systemic Revealing Pharmacological Signalling Pathway Networks in the Hippocampus of Ischaemia-Reperfusion Mice Treated with Baicalin
Source: Evid Based Complement Alternat Med. 2013 Nov 17;2013:630723. doi: 10.1155/2013/630723 (PMC3870072; doi:10.1155/2013/630723)
Supplement: Supplementary file 1 — Supplementary Table 3: Presents the ten most significant biological processes identified by enrichment analysis in the BA and vehicle groups (all P<0.05). Supplementary Table 4: Shows the ten most significant molecular functions in the BA and vehicle groups (all P<0.05). Supplementary Table 5: Presents the ten most significant cellular components associated with the BA and vehicle groups (all P<0.05). Supplementary Table 5A: Presents the ten most statistically significant pathways, based on the MetaCoreTM pathway map analysis (all P<0.05). Supplementary Table 6: Presents the nine networks associated with cerebral ischemia after BA treatment, each with 150 nodes. Supplementary Table 7 and Supplementary Figure 1: Presents the six networks associated with cerebral ischemia in the vehicle group, each with 150 nodes. Supplementary Figure 2: shows that sub network 3 primarily consisted of ZAK, LDB1, TCF12, WIF1, and Kallikrein 1, and that its major functions were intracellular signal transduction and cell surface receptor linked signalling pathway. [file 630723.f1.doc]

**Supplementary Figure 2** Detailed signaling networks diagram of upregulated genes in Baicalin group generated by MetaCore^TM^

Diagrams are shown for (A) VEGFR2/TGFbeta1 (B) p21, (C) SP1 (D) STAT5/cyclinD (E) NF-kB p65 (F) SP1 (G) SP1 (H) SP1 and (I)PAX8/PPARgamma fusion protein

**Supplementary Figure 1** Detailed signaling networks diagrams of upregulated genes in Vehicle group generated by MetaCore^TM^

Diagrams are shown for (A) beta-catenin, (B) p21, (C) C/EBP, (D)SP1/SP3 complex, (E) p53, and (F) SP1.

Supplementary Tables

Supplementary table 3. Network module analysis of selected genes for baicalin

| **#** | **Network** | **GO processes** | **T** | **S** | **P** | **Z** |
| --- | --- | --- | --- | --- | --- | --- |
| 1 | Shc, c-Src, VEGF-A, Paxillin, SMAD3 | regulation of metabolic process (84.7%; 7.498e-47), regulation of cellular process (98.0%; 2.632e-45), enzyme linked receptor protein signaling pathway (39.3%; 1.492e-43), regulation of cellular metabolic process (78.0%; 1.591e-42), regulation of biological process (98.0%; 1.439e-41) | 150 | 18 | 3.470E-28 | 34.67 |
| 2 | G-protein alpha-q, TBP, NR1, RARbeta, c-Src | positive regulation of cellular metabolic process (77.9%; 2.447e-86), positive regulation of metabolic process (78.6%; 4.855e-85), positive regulation of nitrogen compound metabolic process (69.0%; 3.815e-81), positive regulation of nucleobase-containing compound metabolic process (68.3%; 9.910e-81), positive regulation of cellular biosynthetic process (69.7%; 2.913e-80) | 150 | 24 | 1.360E-40 | 46.4 |
| 3 | ZAK, LDB1, TCF12, WIF1, Kallikrein 1 | intracellular signal transduction (55.0%; 3.851e-47), cell surface receptor linked signaling pathway (67.9%; 1.243e-44), positive regulation of cellular process (70.0%; 2.587e-44), positive regulation of biological process (71.4%; 1.573e-42), signaling (80.7%; 3.008e-42) | 150 | 23 | 8.050E-39 | 45.23 |
| 4 | Bcl-XL, E2F1, HDAC1, TFIID, SMAD3 | positive regulation of gene expression (64.8%; 5.099e-78), regulation of macromolecule metabolic process (94.5%; 1.079e-76), positive regulation of macromolecule metabolic process (72.4%; 1.081e-76), regulation of macromolecule biosynthetic process (87.6%; 1.293e-75), regulation of cellular macromolecule biosynthetic process (86.9%; 2.163e-75) | 150 | 21 | 2.930E-34 | 40.54 |
| 5 | E2F3, RARbeta, BAD, STAT6, HGF receptor (Met) | positive regulation of biological process (78.9%; 4.318e-55), positive regulation of cellular process (75.4%; 1.650e-53), regulation of macromolecule metabolic process (83.1%; 7.206e-52), positive regulation of macromolecule metabolic process (59.2%; 1.394e-50), positive regulation of metabolic process (61.3%; 2.362e-50) | 150 | 21 | 3.400E-34 | 40.4 |
| 6 | GAB1, Cyclin A2, MMP-2, Adenosine A1 receptor, MKP-2 | positive regulation of biological process (84.8%; 1.675e-67), positive regulation of cellular process (81.4%; 1.171e-65), cell differentiation (70.3%; 1.579e-53), positive regulation of metabolic process (62.1%; 9.355e-53), positive regulation of cellular metabolic process (60.7%; 1.606e-52) | 150 | 18 | 3.060E-28 | 34.79 |
| 7 | B-Raf, TRAF2, TFIID, CrkL, IL7RA | positive regulation of biological process (77.5%; 1.105e-52), positive regulation of cellular process (73.2%; 5.137e-50), system development (76.1%; 3.496e-49), multicellular organismal development (78.9%; 8.684e-47), regulation of molecular function (63.4%; 9.969e-47) | 150 | 19 | 1.770E-30 | 37.27 |
| 8 | NF-AT2(NFATC1), c-Src, MOGAT1, DGKE, FZD10 | regulation of calcium ion transport via voltage-gated calcium channel activity (20.8%; 1.127e-49), regulation of molecular function (61.7%; 2.548e-46), cell-cell signaling (45.0%; 2.242e-45), multicellular organismal process (87.9%; 3.199e-45), regulation of ion transmembrane transporter activity (21.5%; 6.536e-45) | 150 | 14 | 7.690E-21 | 27.53 |
| 9 | HDAC1, TCF7L1 (TCF3), MeCP2, TBP, c-Src | positive regulation of macromolecule metabolic process (89.6%; 7.417e-55), positive regulation of RNA metabolic process (79.1%; 7.353e-53), positive regulation of transcription, DNA-dependent (77.6%; 3.780e-52), positive regulation of metabolic process (89.6%; 4.309e-52), positive regulation of gene expression (79.1%; 5.570e-52) | 68 | 9 | 4.590E-15 | 25.87 |

| T: Total nodes; | S: Seed nodes; | P: P value; | Z: Z score. |
| --- | --- | --- | --- |

**Supplementary table 2**. Network module analysis of selected genes for vehicle

| **#** | **Network** | **GO processes** | **T** | **S** | **P** | **Z** |
| --- | --- | --- | --- | --- | --- | --- |
| 1 | G-protein alpha-q, TFIID, ARHGEF1 (p115RhoGEF), Shc, E2F3 | positive regulation of molecular function (46.0%; 7.330e-37), developmental process (72.7%; 1.579e-35), positive regulation of cellular process (62.7%; 3.878e-35), multicellular organismal development (69.3%; 1.115e-34), cellular response to stimulus (78.0%; 1.517e-34) | 150 | 20 | 1.480E-33 | 41.45 |
| 2 | Bcl-XL, HDAC1, NF-AT2(NFATC1), GAK, MKP-2 | positive regulation of RNA metabolic process (57.1%; 2.075e-60), positive regulation of transcription, DNA-dependent (55.7%; 2.684e-59), regulation of transcription from RNA polymerase II promoter (58.6%; 1.257e-58), positive regulation of transcription from RNA polymerase II promoter (49.3%; 6.389e-58), positive regulation of gene expression (55.7%; 2.132e-56) | 142 | 20 | 3.980E-34 | 42.78 |
| 3 | TBP, STAT6, c-Src, JunB, JunD | positive regulation of RNA metabolic process (53.3%; 3.425e-57), positive regulation of transcription, DNA-dependent (52.0%; 3.371e-56), regulation of transcription from RNA polymerase II promoter (54.7%; 2.595e-55), positive regulation of cellular process (75.3%; 8.149e-55), positive regulation of nitrogen compound metabolic process (54.7%; 1.662e-53) | 150 | 19 | 1.640E-31 | 39.49 |
| 4 | TRAF2, KCNQ1, TRADD, Caspase-2, HTR2C (HTR1C) | purine nucleotide metabolic process (32.2%; 8.120e-32), nucleotide metabolic process (34.2%; 9.017e-32), nucleoside phosphate metabolic process (34.2%; 9.017e-32), nucleobase-containing small molecule metabolic process (34.9%; 1.714e-31), heterocyclic metabolic process (36.2%; 1.385e-30) | 150 | 18 | 1.500E-29 | 37.64 |
| 5 | Axin1, IKK-gamma, NR1, IL-1 alpha, MKP-2 | positive regulation of biological process (68.8%; 8.611e-38), positive regulation of cellular process (65.2%; 1.649e-36), regulation of cellular metabolic process (75.2%; 2.248e-35), negative regulation of biological process (63.8%; 2.521e-35), regulation of primary metabolic process (73.8%; 5.606e-34) | 150 | 13 | 8.670E-20 | 27.35 |
| 6 | Shc, c-Src, HGF receptor (Met), TAFII55, MOGAT1 | translational initiation (21.1%; 1.688e-36), viral genome expression (17.7%; 9.152e-36), viral transcription (17.7%; 9.152e-36), translational termination (17.7%; 6.115e-35), SRP-dependent cotranslational protein targeting to membrane (17.7%; 8.697e-33) | 150 | 9 | 1.700E-12 | 18.58 |

| ***Note***: T: Total nodes; | S: Seed nodes; | P: P value; | Z: Z score. |
| --- | --- | --- | --- |

**Supplementary Table 4.** Biological processes associated with the selected genes in order of significance

| # | Process | Total | In data | *P*-value |
| --- | --- | --- | --- | --- |
| **Baicalin** | | | | |
| 1 | Positive regulation of cellular process* | 3704 | 95 | 9.195E-44 |
| 2 | Positive regulation of biological process | 4086 | 98 | 3.379E-43 |
| 3 | Signaling* | 5682 | 110 | 1.595E-42 |
| 4 | Cell communication | 5855 | 110 | 3.467E-41 |
| 5 | Signal transduction* | 5179 | 104 | 5.407E-40 |
| 6 | Positive regulation of molecular function* | 1654 | 66 | 1.747E-38 |
| 7 | Regulation of phosphorylation | 1139 | 57 | 8.972E-38 |
| 8 | Regulation of protein phosphorylation | 1050 | 55 | 2.435E-37 |
| 9 | Regulation of phosphorus metabolic process | 1234 | 58 | 5.141E-37 |
| 10 | Regulation of phosphate metabolic process | 1234 | 58 | 5.141E-37 |
| **Vehicle** | | | | |
| 1 | Positive regulation of catalytic activity | 1445 | 53 | 2.329E-33 |
| 2 | Positive regulation of molecular function* | 1701 | 55 | 5.436E-32 |
| 3 | Regulation of catalytic activity | 2306 | 60 | 3.158E-30 |
| 4 | Positive regulation of hydrolase activity | 828 | 41 | 3.894E-30 |
| 5 | Regulation of molecular function | 2794 | 64 | 1.611E-29 |
| 6 | Positive regulation of cellular process* | 3822 | 72 | 6.337E-29 |
| 7 | Signal transduction* | 5260 | 82 | 6.683E-29 |
| 8 | Signaling* | 5768 | 85 | 8.617E-29 |
| 9 | Cellular response to stimulus | 6514 | 89 | 1.476E-28 |
| 10 | Intracellular signal transduction | 1757 | 52 | 3.989E-28 |

* biological processes common to both baicalin and vehicle groups are shown in red.

Supplementary Table 5. Molecular functions associated with the selected genes in order of significance

| # | Function | Total | In data | P value |
| --- | --- | --- | --- | --- |
| **Baicalin** | | | | |
| 1 | Protein binding* | 8288 | 87 | 8.578E-23 |
| 2 | Enzyme binding* | 1179 | 29 | 5.343E-14 |
| 3 | Binding* | 13578 | 95 | 9.462E-13 |
| 4 | Protein kinase activity | 685 | 21 | 4.943E-12 |
| 5 | Signal transducer activity* | 2157 | 35 | 1.322E-11 |
| 6 | Molecular transducer activity* | 2157 | 35 | 1.322E-11 |
| 7 | Phosphotransferase activity, alcohol group as acceptor | 807 | 22 | 1.416E-11 |
| 8 | Kinase binding* | 463 | 17 | 4.159E-11 |
| 9 | Kinase activity | 944 | 23 | 4.301E-11 |
| 10 | Protein kinase binding* | 406 | 16 | 5.797E-11 |
| **Vehicle** | | | | |
| 1 | Protein binding* | 8381 | 74 | 8.95E-20 |
| 2 | Binding* | 13673 | 80 | 1.04E-10 |
| 3 | Enzyme binding* | 1211 | 22 | 8.40E-10 |
| 4 | Kinase binding* | 473 | 14 | 3.76E-09 |
| 5 | Transferase activity, transferring P-containing groups | 1090 | 19 | 2.87E-08 |
| 6 | Protein kinase binding* | 412 | 12 | 6.42E-08 |
| 7 | Signal transducer activity* | 2138 | 26 | 8.00E-08 |
| 8 | Molecular transducer activity* | 2138 | 26 | 8.00E-08 |
| 9 | BH domain binding | 13 | 4 | 1.47E-07 |
| 10 | Enzyme activator activity | 458 | 12 | 2.01E-07 |

* molecular functions common to both baicalin and vehicle groups

Supplementary Table 6. Cellular components associated with the selected genes

| # | Localization | Total | In data | P value |
| --- | --- | --- | --- | --- |
| **Baicalin** | | | | |
| 1 | Postsynaptic density* | 143 | 8 | 3.720E-06 |
| 2 | Dendritic spine head* | 143 | 8 | 3.720E-06 |
| 3 | Cytosol* | 2490 | 34 | 1.029E-05 |
| 4 | Neuron spine* | 197 | 8 | 3.832E-05 |
| 5 | Dendritic spine* | 197 | 8 | 3.832E-05 |
| 6 | Cytoplasm | 11101 | 93 | 4.297E-05 |
| 7 | Transcription factor complex | 359 | 10 | 1.012E-04 |
| 8 | Heterotrimeric G-protein complex* | 40 | 4 | 1.193E-04 |
| 9 | Internal side of plasma membrane* | 121 | 6 | 1.243E-04 |
| 10 | Chromatin | 302 | 9 | 1.371E-04 |
| **Vehicle** | | | | |
| 1 | Cytosol* | 2435 | 34 | 6.40E-08 |
| 2 | Postsynaptic density* | 142 | 8 | 9.03E-07 |
| 3 | Dendritic spine head* | 142 | 8 | 9.03E-07 |
| 4 | Delta DNA polymerase complex | 5 | 3 | 1.44E-06 |
| 5 | Heterotrimeric G-protein complex* | 40 | 5 | 2.18E-06 |
| 6 | Internal side of plasma membrane* | 121 | 7 | 3.75E-06 |
| 7 | Nuclear chromosome part | 256 | 9 | 9.09E-06 |
| 8 | Extrinsic to internal side of plasma membrane | 55 | 5 | 1.08E-05 |
| 9 | Neuron spine* | 199 | 8 | 1.11E-05 |
| 10 | Dendritic spine* | 199 | 8 | 1.11E-05 |

*cellular components common to both baicalin and vehicle groups

Supplementary Table 1A GeneGo pathway map distribution of selected genes of BA

| **#** | **Maps** | **Total** | **In Data** | **pValue** |
| --- | --- | --- | --- | --- |
| 1 | Cytoskeleton remodeling_TGF, WNT and cytoskeletal remodeling | 111 | 13 | 4.322E-11 |
| 2 | Development_Flt3 signaling | 44 | 8 | 8.367E-09 |
| 3 | Neurophysiological process_NMDA-dependent postsynaptic long-term potentiation in CA1 hippocampal neurons | 80 | 9 | 7.386E-08 |
| 4 | G-protein signaling_Regulation of p38 and JNK signaling mediated by G-proteins | 39 | 7 | 8.309E-08 |
| 5 | Development_VEGF signaling via VEGFR2 - generic cascades | 84 | 9 | 1.136E-07 |
| 6 | G-protein signaling_G-Protein alpha-i signaling cascades | 27 | 6 | 1.910E-07 |
| 7 | Development_TGF-beta-dependent induction of EMT via MAPK | 47 | 7 | 3.189E-07 |
| 8 | Transcription_Transcription factor Tubby signaling pathways | 17 | 5 | 4.634E-07 |
| 9 | Cytoskeleton remodeling_Cytoskeleton remodeling | 102 | 9 | 6.123E-07 |
| 10 | Normal and pathological TGF-beta-mediated regulation of cell proliferation | 33 | 6 | 6.819E-07 |

Table 1B GeneGo pathway map distribution of selected genes of vehicle

| **#** | **Maps** | **Total** | **In Data** | **pValue** |
| --- | --- | --- | --- | --- |
| 1 | Development_G-Proteins mediated regulation MAPK-ERK signaling | 46 | 7 | 6.62E-08 |
| 2 | Reproduction_GnRH signaling | 72 | 8 | 9.12E-08 |
| 3 | Neurophysiological process_NMDA-dependent postsynaptic long-term potentiation in CA1 hippocampal neurons | 80 | 8 | 2.10E-07 |
| 4 | Development_Thyroliberin signaling | 61 | 7 | 4.91E-07 |
| 5 | G-protein signaling_Regulation of p38 and JNK signaling mediated by G-proteins | 39 | 6 | 5.72E-07 |
| 6 | Apoptosis and survival_TNFR1 signaling pathway | 43 | 6 | 1.04E-06 |
| 7 | Development_Flt3 signaling | 44 | 6 | 1.20E-06 |
| 8 | Development_PIP3 signaling in cardiac myocytes | 47 | 6 | 1.79E-06 |
| 9 | G-protein signaling_G-Protein alpha-i signaling cascades | 27 | 5 | 2.04E-06 |
| 10 | Signal transduction_PKA signaling | 51 | 6 | 2.93E-06 |
